# Dislocation of the Astragalus and Reduction

**Published:** 1846-10

**Authors:** P. M. Hosking

**Affiliations:** Sydney, New South Wales


*Dislocation of the Astragalus and reduction.* By P. M. Hosking, M. D., M.R.C.S., Sydney, New South Wales.—Accidents to the ankle-joint are of unfrequent occurrence, where no dislocation or fracture takes place; but having met with a case of dislocation of the astragalus very recently, which I reduced with perfect ease, and complete recovery to the patient; and being also aware of the difficulties presenting themselves generally in the different modes recommended and adopted by "authorities," I am induced to record this case in your far-read publication, especially as I perceive in the Lancet, for October, 1844, pp. 35, 70, a paper on dislocations of the astragalus, read before the Royal Medical and Chirurgical Society of London, and that in the case there cited in illustration much difficulty seems to have occurred, and fracture of the fibula took place at the time of the accident, with displacement of the tibia. This case is as follows:

C. B., a young man, aged twenty-six, jumped from a door down three or four steps, and fell to the ground. I was sent for immediately, and found him supported by some men. Upon examination, I found the left foot dislocated, and turned inwards and upwards, with a lump in the back of the foot, or instep, shining and glossy under the skin; the fibula and tibia were in situ, and no fracture existed. I had him removed up stairs on a bed, and then saw that the astragalus had been thrown forwards out of its place upon the dorsum of the foot. I desired one of the men to hold him by the knee; and the patient being faint, I made extension at the same time that I pressed with my two thumbs upon the displaced bone, having the palms of both hands under the os calcis, which immediately slipped into its place, and perfectly restored the shape of the foot and ankle. I then put a roller tightly around it, ordered a cold lotion, desired him to keep quiet, and left him. Upon examination the next day, I found a slight wound of the skin, just over where the bone lay; I put splints slightly on it, and continued the lotion. On the fifth day, he was out, without my permission, on a crutch; on the seventh day, he complained of cold in the limb, which was still swollen. I removed the splints and calico roller, and applied a roller of flannel. There was no difficulty in the reduction, and the patient recovered without the occurrence of any bad symptoms.—*Lancet.*

